# Reviewers Index

**DOI:** 10.3400/avd.ri.22-01000

**Published:** 2022-12-25

(The reviewers who completed the peer-review for manuscripts submitted during Nov 1, 2021–Oct 31, 2022.)

The thirteen reviewers written in boldface reviewed more than 5 papers.

Annals of Vascular Diseases do thank all the reviewers for their strong support and cooperation.

## A

Adachi, Koichi

Akasaka, Kazumi

Akutsu, Koichi

Al-Salman, Mussaad

**Altarazi, Louay**

Alzahrani, Hasan Ali

**Anai, Hiroshi**

Aoki, Atsushi

Aoyagi, Shigeaki

Asakura, Toshihisa

Asano, Soichi

## B

**Banno, Hiroshi**

**Bashar, Abul Hasan**

Bessho, Ryuzo

## C

**Cheng, Stephen**

## D

Deguchi, Juno

## F

Fletcher, John

Fujii, Takeshiro

Fujimura, Naoki

Fukuda, Hirotsugu

**Fukuda, Ikuo**

Fukui, Kozo

Furukawa, Kojiro

**Furuyama, Tadashi**

## H

Hamamoto, Masaki

Handa, Nobuhiro

Harada, Hirohisa

Haruta, Naoki

Hashizume, Kenichi

**Hattori, Tsutomu**

Hiramatsu, Yuji

Hiraoka, Arudo

Hirokawa, Masayuki

Hiromatsu, Shinichi

Hosaka, Akihiro

Hosaka, Junro

## I

Ichihashi, Shigeo

Ihara, Tsutomu

**Iida, Yasunori**

Imai, Takahiro

**Inaba, Masashi**

Inoue, Masanori

Inuzuka, Kazunori

Ishibashi, Hiroyuki

Ishikawa, Tetsuya

**Ito, Hiroyuki**

Itoi, Toshiyuki

Iwata, Hirohide

Iyori, Keiji

Izumi, Yuichi

## J

Jibiki, Masatoshi

Jin, Hyun Joh

## K

**Kadohama, Takayuki**

Kamal, Dhafer

Kamiya, Hiroyuki

Kanaoka, Yuji

Kato, Masaaki

Katsumata, Takahiro

Kawaharada, Nobuyoshi

Khan, Mohammad

Kitagawa, Takeshi

Kodama, Akio

Koizumi, Jun

Kokubo, Taku

Komai, Hiroyoshi

Kondo, Norihiro

Kritayakirana, Kritaya

Kudo, Toshifumi

Kuma, Sosei

Kumakura, Hisao

Kurimoto, Yoshihiko

## M

Maeda, Hideaki

**Maeda, Koji**

Masaki, Hisao

Matsubara, Junichi

Matsubara, Kentaro

Matsubara, Shinobu

Matsuda, Hitoshi

Matsumoto, Takuya

Matsuo, Hiroshi

Matsushita, Masahiro

Matsuyama, Katsuhiko

Midorikawa, Hirofumi

Minatoya, Kenji

Mo, Makoto

Morikage, Noriyasu

Morota, Tetsuro

Mukohara, Nobuhiko

Murakami, Atsubumi

## N

Nakamura, Takashi

Nakazawa, Tatsu

Nishibe, Toshiya

Nishigami, Kazuhiro

Nishimura, Kengo

Nishimura, Motonobu

Nishimura, Yoshiharu

Nunokawa, Masao

## O

Obara, Hideaki

Obitsu, Yukio

Ogawa, Yukihisa

Ohtake, Hiroshi

Okada, Kenji

Okazaki, Jin

Orihashi, Kazumasa

Otani, Norifumi

## P

Pinjala, Ramakrishna

## S

Saiga, Atsushi

Sakuda, Hitoshi

Sato, Osamu

Satokawa, Hirono

Sawano, Makoto

Shigematsu, Kunihiro

Shindo, Shunya

Shirasugi, Nozomu

Sugano, Norihide

Sugimoto, Takaki

Sumi, Makoto

Suzuki, Shinichi

## T

Taguchi, Shinichi

Takagi, Yasushi

Takahashi, Toshiki

Takayama, Toshio

Takayama, Yutaka

Takekawa, Shoichi

Tanemoto, Kazuo

Tateno, Kaoru

Tayebi, Pouya

Ting, Albert

Tomaru, Takanobu

Tsuda, Kazumasa

Tsukube, Takuro

Tsutsumi, Koji

## U

Uchida, Keiji

Ueyama, Keishi

Unno, Naoki

## V

Vardhan, Vivek

## W

Wada, Hideichi

Wang, Jinsong

Washiyama, Naoki

## Y

Yamaguchi, Atsushi

Yamamoto, Hiroshi

Yamamoto, Kota

Yamamoto, Naoto

Yamamoto, Takeshi

Yamamura, Mitsuhiro

Yasugi, Takumi

Yiu, Wai-ki

Yokoyama, Naoyuki

Yoshitake, Akihiro

Yoshizumi, Masao

Yunoki, Yasuhiro

## Z

Zempo, Nobuya

